# Carbon footprint and embodied nutrition evaluation of 388 recipes

**DOI:** 10.1038/s41597-023-02702-1

**Published:** 2023-11-10

**Authors:** Yin Long, Liqiao Huang, Rinakira Fujie, Pan He, Zhiheng Chen, Xiaoyan Xu, Yoshikuni Yoshida

**Affiliations:** 1 Graduate School of Engineering, University of Tokyo, Tokyo, Japan; 2 School of Earth and Environmental Sciences, Cardiff University, Cardiff, UK

**Keywords:** Climate-change adaptation, Nutrition, Climate-change mitigation

## Abstract

Food consumption, which delivers fundamental energy and essential nutrients to human beings, is crucial for achieving a series of sustainable goals. Alongside rising population growth and living standards, there has been a significant increase in food cultivation demands, supply chain complexities, and waste management. Therefore, to protect human health and the environment, promoting sustainable food systems and the uptake of sustainable dietary habits are vital. Yet, information on the environmental and health impact of dietary choices remains inconsistent across multiple evaluation methods, which fail to deliver essential ideas to consumers. In this study, we formulate an integrated approach using Environmentally Extended Input-Output analysis, covering the food supply chain from production to the distribution phase, complemented with a hybrid Life Cycle Assessment for cooking and disposal processes, to quantify the carbon footprint of specific recipes. Our dataset also includes the distinct nutritional values of each recipe. This dataset not only informs the food industry and recipe platforms, enabling more sustainable choices, but also helps individuals balance nutritional value with environmental impact, leading to more informed and sustainable dietary decisions.

## Background & Summary

The global food system significantly contributes to numerous environmental crises, including climate change, deforestation, and water scarcity, and also presents numerous nutritional and health challenges. Specifically, it is responsible for 19–29% of total anthropogenic greenhouse gas (GHG) emissions, more than 70% of surface and groundwater usage^1^, and 37% of the Earth’s landmass occupation^2^. On the other hand, the consumption of different food products has a marked influence on the public health of each individual. For example, due to the unbalanced food supply and the global population burgeoning, a considerable portion of the global population faces malnutrition issues, ranging from excessive intake of sugar, trans fat, and red and processed meat to deficiencies in essential nutrients like vegetables, fruits, and whole grains^3–7^. This imbalance contributes to overweight or obesity in one-third of the global population, while inadequate micronutrient intake affects 2 billion people^8,9^, leading to a host of non-communicable diseases such as diabetes, stroke, and heart disease^10^. Notably, dietary risks were the leading cause of global disability-adjusted life-years (DALYs) from 1990 to 2013^8^. Therefore, to foster a Net Zero and more sustainable future, it is imperative to stimulate demand-end changes and transition consumer dietary patterns, taking into account both environmental and health perspectives.

In response, scholars and policymakers have delved into the environmental and health outcomes of diverse global dietary patterns, identifying synergies that benefit human health and the environment at multiple levels. At the global level, scenario analyses have shown the potential benefits of reducing red meat consumption, which include not only improved public health but also a reduction in GHG emissions, water use, eutrophication, and other environmental impacts within planetary boundaries^11–16^. On the other hand, country-specific cases aim for more precise and granular analysis, focusing on the household or individual level. These studies explore sub-national socio-economic heterogeneity and involve discussions about environmental and health justice^17–20^. While these investigations establish general principles for healthy and sustainable food consumption, there remains a need for more accurate data on the environmental and nutritional impacts at the food-product level to guide consumer behavior^7,21^. However, the data’s accuracy can still be compromised by culinary practices, as consumers combine food products as ingredients and adopt various cooking methods.

For the purpose of providing detailed diet information to consumers with their real-world dietary impacts, recent studies start noticing the importance of dish-level analysis^22–24^. For instance, scholars began leveraging aggregated scores to examine diets’ nutritional elements at the dish level, providing more precise nutrient intake estimates across varied dietary choices and patterns^25–27^. Simultaneously, the sustainable diet is also gaining attention in addition to the nutrition aspect. With the establishment of online recipe repositories, carbon footprint calculations for individual recipes are becoming more diverse and accessible^22,28–31^. Moreover, environmental footprint analyses for recipes provided revelations on how ingredient substitutions, particularly with locally sourced foods, can significantly attenuate associated carbon footprints^23,32^. Despite these efforts, there is still a conspicuous gap in our collective understanding when it comes to the integrated assessment of both environmental and nutritional outcomes of dishes in existing research. In the real world, consumers do not consume food items directly, the majority convert food ingredients into different dishes and eat. Therefore, to provide consumers with understandable information, there is an urgent need for dish-level evaluation of the interconnected nutritional and environmental impacts to motivate a culinary behavioral change.

To bridge this research gap, our dataset encompasses the environmental footprints, involving both the ingredients and the cooking methods, and the nutritional quality of recipes, as part of a broader endeavor to understand and promote sustainable dietary patterns. It should be noted that existing research generally adopts the Life Cycle Assessment (LCA) methodology in the context of calculating the carbon footprint of recipes^7,11,33–35^. Nevertheless, the research methodology involved in the establishment of this dataset is the Environmentally Extended Input-Output (EEIO) approach, a robust tool for assessing the environmental impacts of product systems^36,37^. Though both LCA and EEIO each have their unique application areas and strengths, compared to the LCA method requiring the scope definition, the EEIO method offers a comprehensive and systemic perspective that encompasses all stages within the entire supply chain^38^. Additionally, EEIO is based on extensive economic data and industrial statistics at global, national, or regional levels, ensuring high traceability and accuracy, making it outperforms in terms of comparability and standardization of results^39,40^. Hence, this method allowed us to quantify the life-cycle GHG emissions for each recipe, capturing both the ingredients and the cooking process involved.

Moreover, our dataset encompasses the nutrition as well as environmental footprints of popular recipes from Ajinomoto Co., which is a multinational corporation that specializes in food and amino science, producing seasonings, oils, and other food products. As noted, cultural traditions profoundly shape dietary habits, dictating food selection, preparation methods, meal times, dietary practices, and attitudes toward food^29,41–44^. In contrast to previous research that mainly concentrates on cuisines in Western countries, our research sheds light on dishes consumed by the Japanese population. It should be noted that Japan’s traditional dietary patterns, defined by high consumption of seafood, rice, vegetables, and a relatively low intake of red meat, have long been recognized for their health benefits^45–47^. However, these traditional practices are affected by a marked trend towards westernized dietary habits, characterized by a higher consumption of processed and fast foods^45,48–50^. Consequently, our dataset includes a broader range of recipes, covering not only traditional Japanese dishes but also dishes from other countries. This comprehensive coverage offers insights that may inform sustainable food policies not only in Japan but potentially in other nations grappling with issues of sustainable dietary transition as well.

## Method

Since consumers typically consume dishes, rather than raw ingredients, this dataset recognizes the growing importance of dish-level analysis to bridge the research gap of analyzing diet by ingredients only. Hence, our focus is to establish a dataset to cover both the environmental outcomes and nutritional content of recipes. To achieve this, our dataset encompasses the environmental footprint, inclusive of ingredients and cooking methods, along with the nutritional quality of recipes as part of broader efforts to understand and promote sustainable dietary patterns, with each step of the dataset establishment presented in the **Method** section.

### Recipe data collection

To collect the data necessary for this study related to various dishes, we used the recipe site “Recipe Encyclopedia” operated by Ajinomoto Co., Inc., one of the largest food companies based in Japan^51^. The online recipes dataset provides a wide range of recipes, and the information related to each recipe is also detailed, hence it was chosen as the source of data collection for each dish. Therefore, by collecting the information from the Recipe Encyclopedia of Ajinomoto we conducted a search using the names of ingredients as categorized by Ajinomoto Co., Inc. as indicated in Table 1, and the resulting recipes were added to the collection candidate list in the order of their recentness. Information in this table provides clarity on ingredients that were used to compile the recipe dataset.

**Table 1:** Ingredient for searching recipes.

Animal-Derived Products	Meat	Beef
		Pork
		Pork Belly
		Chicken
		Chicken Thigh
		Chicken Wing Tip
		Ground Meat
		Mixed Ground Meat
		Wiener/Sausage
		Ham
	Fish & Seafood	Tuna
		Canned Tuna
		Mackerel
		Canned Mackerel
		Salmon
		Cod
		Squid
		Shrimp
		Octopus
		Sardine
		Horse Mackerel
	Others	Eggs
Plant-Derived Products	Vegetables	Cabbage
		Onion
		Carrot
		Bean Sprouts
		Potato
		Bell Pepper
		Broccoli
		Shimeji Mushrooms
		Shiitake Mushrooms
		Eryngii (King Oyster Mushroom)
		Pumpkin
		Cucumber
		Radish
		Eggplant
		Chinese Cabbage
		Bamboo Shoots
	Grains & Grain Products	Udon Noodles
		Soba Noodles
		Somen Noodles
		Pasta
	Legumes & Legume Products	Tofu
		Fried Tofu
	Others	Hiziki (a type of seaweed)
		Wakame (a type of seaweed)
		Soymilk

**Fig. 1:**
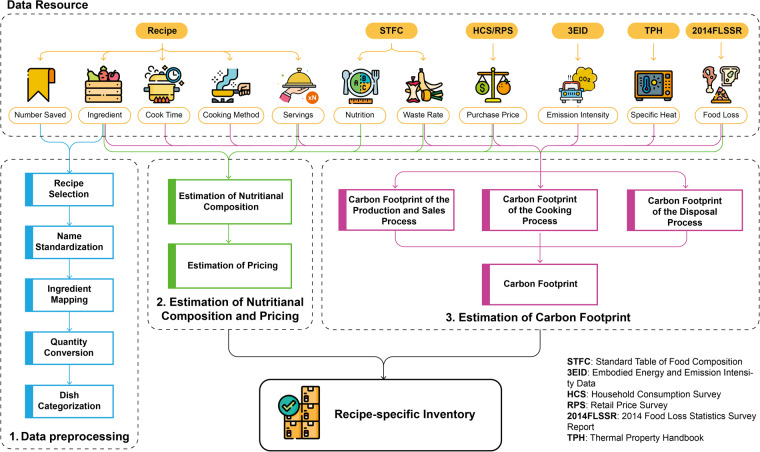
Flowchart of this dataset.

The number of non-duplicate recipe data collected in this manner is 10,337. As of May 9, 2022, when the recipe data was collected, the total number of recipes recorded in the “Recipe Encyclopedia” was 11,956, with the collected recipe data accounting for 86.5% of all recorded recipes. We have deleted very similar recipes. The general flow chart of this dataset is shown in Fig. 1, and the collected recipe data then underwent the following preprocessing steps with details:

### Step 1. Recipe Selection

Since the focus of this study is on representative dishes and the most popular recipes within the collected recipes, we extracted recipes from the “Recipe Encyclopedia” that had received over 1,000 saves by users as of May 9, 2022. We added rice recipes to these extracted recipes, and a total of 388 recipes were targeted for this study. The information pertaining to each recipe along with corresponding descriptive details in this database is presented in Table 2.

**Table 2:** Information Archive for Selected Recipes from the Recipe Encyclopedia.

Information obtained from each recipe	Description
Recipe ID	A unique 6-digit number for each recipe for identification purposes
Recipe Name	Name of the recipe (used exactly as expressed on the “Recipe Encyclopedia”)
Published Datetime	Time when the recipe was posted
Modified Datetime	Time when the recipe was last updated/modified
Servings	Number of servings for the recipe
Cook Time	Preparation and cooking time for the recipe (excluding marination and similar steps)
Ingredients	Ingredients used in the recipe and their quantities
Directions	Cooking instructions, preparation steps
Cuisine Style	Type of cuisine (Japanese, Western, Chinese, Ethnic, etc.)
Cuisine Type	Classification of the dish (main dish, side dish, soup, etc.)
Number Saved	Number of times the recipe was saved

### Step 2. Ingredient name standardization

Ingredient names were standardized for uniformity, omitting any part of the name that indicated specific products, such as initial English letters or ‘AJINOMOTO’. This standardization process was conducted on all ingredients utilized in the 388 recipes. After removing duplicates from the ingredient list after processing, we ultimately acquired 349 unique ingredients.

### Step 3. Ingredient mapping

For each of these 349 ingredients, they were matched to the most similar item in the Standard Tables of Food Composition in Japan^52^. The Standard Tables of Food Composition in Japan (2015, Seventh Revised Edition) provides a comprehensive categorization of 2191 foods into various groups like cereals, vegetables, meats, and more. The tables consider foods in their raw, cooked, and processed states, including traditional Japanese cuisine. Each food item is analyzed for its nutrient components, including proteins, lipids, carbohydrates, vitamins, and minerals, providing a detailed overview of nutritional values per 100 g of edible portion, and energy values are calculated using energy conversion factors. This meticulous categorization and analysis make these tables a vital resource for dietary planning and analysis. In this research, we undertook a comprehensive analysis of various nutrients. Primary among these are the macronutrients, encompassing Energy (kcal), Carbohydrates (g), Protein (g), and Fat (g). Subsequently, we evaluated vitamins, specifically Vitamin A (μg), Vitamin C (mg), and Vitamin E (mg). In the realm of minerals, we assessed the concentrations of Zinc (mg), Calcium (mg), Iron (mg), Potassium (mg), Magnesium (mg), Folic Acid (μg), and Dietary Fiber (mg). Additionally, other parameters, including Saturated Fats (g), Cholesterol (mg), and Salt Equivalent (g), were also incorporated into our analysis.

### Step 4. Ingredient quantity conversion

The quantities of ingredients listed in each recipe were converted into a calculable form. While certain quantities, especially for water and seasoning, were indicated in milliliters (ml), these were converted to grams (g). For small quantities such as “appropriate amount,” “a bit,” “a pinch,” etc., these were uniformly considered as 0 g. For expressions unique to ingredients, like “1 carrot,” “2 eggs,” etc., we used the “Estimated Quantity of Ingredients” table from the Ajinomoto site for conversion to grams.

### Step 5. Dish categorization

The primary objective in categorizing the dishes is to systematically analyze the environmental impact of individual ingredients, which can significantly differ based on their origin and production methods. For each dish, based on the ingredients used, we classified the dishes under the following rules. The first rationale behind our categorization is for dishes with ingredients such as beef, pork, chicken, minced meat, other fresh meat, and processed meat, which are widely recognized to have considerable environmental footprints. Notably, among the dishes targeted in this study, there were no dishes that used multiple types of the aforementioned meat or seafood simultaneously. Thus, dishes using the corresponding ingredients were individually classified corresponding to the ingredient used.

The second rule was applied to grains, eggs, vegetables, mushrooms, and beans. Given the varied environmental impacts between plant-derived ingredients, this rule aims to differentiate dishes based on their primary plant ingredient. For dishes that do not use meat or seafood as ingredients, they were classified according to the type of ingredient (grains, eggs, vegetables, mushrooms, beans) that was used in the largest quantity.

### Estimation of Nutritional Composition and Pricing by Recipe-specific

The estimation of nutritional composition was conducted using the weight of each ingredient used in the recipe and the nutritional composition per unit weight of each ingredient. For each ingredient in the recipe, the items correspond to the Japan-Standard Tables of Food Composition. Also, considering the parts that are removed and discarded during preprocessing, such as skin, bones, and shells of seafood, eggshells, and cores of vegetables, we only calculated the nutrients that can be taken from the edible part. The weight of the edible part was calculated using the waste rate listed in the Japan-Standard Tables of Food Composition applied to the amount listed in the recipe. In the calculation of the nutritional composition, we did not consider waste other than inedible parts as mentioned in Section 2. Also, we ignored the loss or denaturation of nutrients caused by cooking and heating. The nutrient intake per serving from each recipe can be represented by the following Eq. 1.

1 $${{\rm{N}}}_{{\rm{j}}}=\frac{{\sum }_{{\rm{i}}}{{\rm{Q}}}_{{\rm{i}}}\times \left(1-{{\rm{D}}}_{{\rm{i}}}\right)\times {{\rm{N}}}_{{\rm{ij}}}}{{\rm{S}}}$$

Here, N_j_ represents the amount of nutrient j that can be taken when one serving of a certain dish is eaten. Q_i_ represents the weight listed in the recipe of ingredient i used in this dish [g]. D_i_ is the waste rate of ingredient i, and N_ij_ is the content of nutrient j per 1 g of the edible part of ingredient i. For unmeasured nutrients and trace nutrients, we calculated them as 0. S is the number of servings per recipe.

**Fig. 2:**
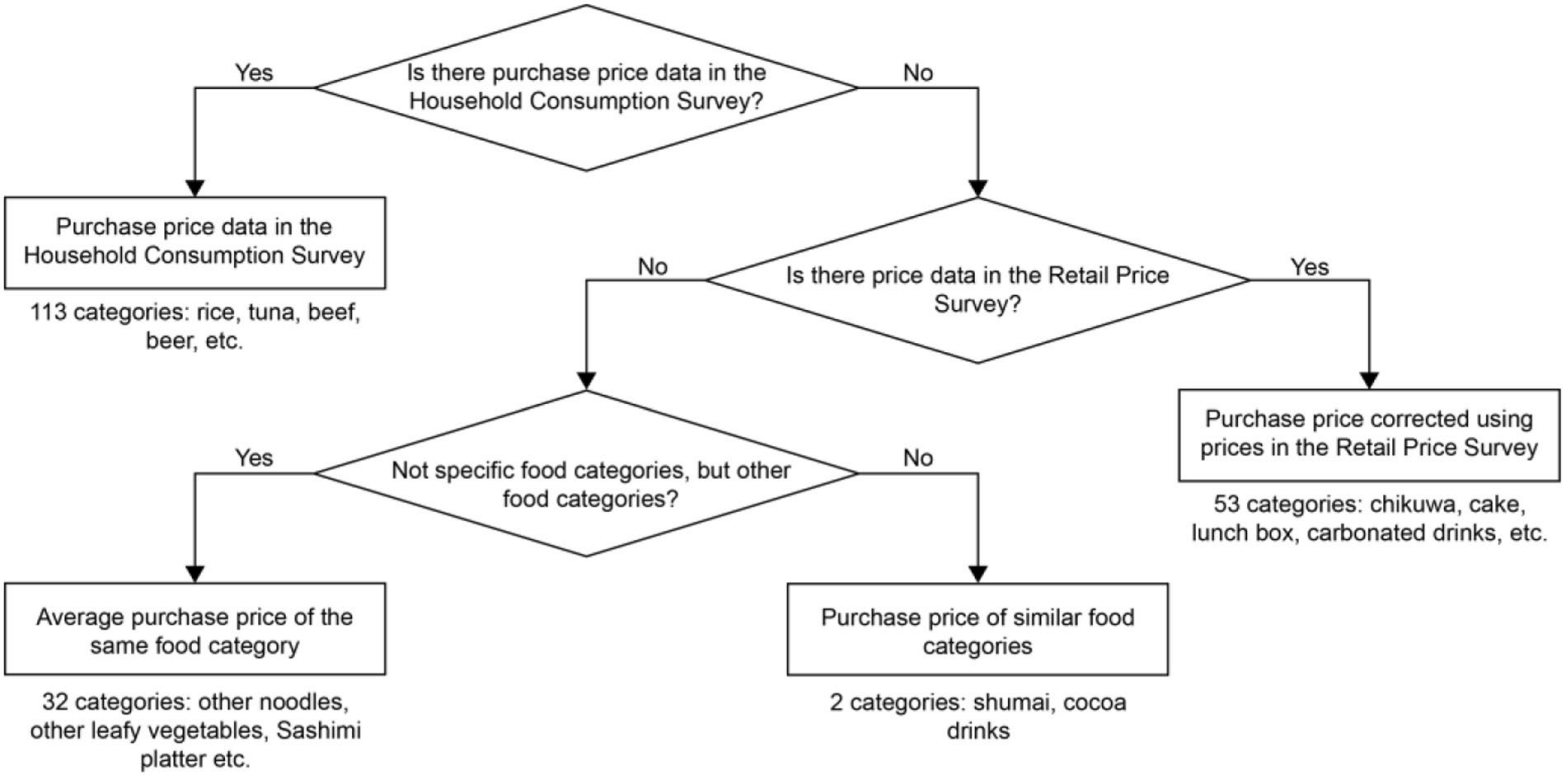
Flowchart of the unit price of food items purchased in this dataset.

Next, regarding the price per serving for each dish, we calculated only the costs necessary to purchase the ingredients used in the dish. To note, costs associated with the storage, cooking, and disposal of ingredients are not included in the price of the recipes in the current study. Moreover, we used the household consumption survey from the Family Income and Expenditure Survey by the Statistics Bureau of Japan to obtain data on household consumption patterns to accurately assess the carbon footprint of food^53^. This data shed light on the average monthly expenditure on various food items procured by Japanese households as well as their unit prices, which is integral to our evaluation process, enabling us to correlate price information with recipe ingredients, so as to conduct our carbon footprint assessments of the 388 recipes. Figure 2 presents the flowchart of this calculation. However, in calculating the purchase price of each food item, since the household survey does not conduct surveys on the purchase price for one-person households, we used data from households of two or more. In addition, due to differences in the standard physical unit across food items in the purchase price data we used, we converted all data to a price per 100 g. During this process, milliliters, as used in condiments and beverages, were uniformly approximated as 1 g. The household survey does not distinguish between domestic and imported goods; therefore, the purchase prices for each food item used in this study include both domestic and imported goods.

As illustrated in Fig. 2, for some food items lacking purchase price data in the household survey, we calculated these using price information from the 2015 retail price statistics survey^54^. This method of purchase price calculation was applied to 53 food items, such as “chikuwa” and “cake”. Below, we explain the specific calculation method. In the retail price statistical survey, considering representativeness, we targeted prefectural capitals and cities with populations of 150,000 or more. Based on the average annual retail price and the weight of surveyed items in each city, we calculated the price per 100 g and took this as the relevant item’s price. However, the retail price statistics survey aims to follow the price changes of specific products for each food item, and there could be differences in the purchase prices in the household survey. Therefore, we considered the difference between prices and purchase prices and performed a correction for the prices. The correction was calculated as a ratio of the average purchase price in the household survey and the price in the retail price statistics survey for items listed in both surveys and the average of this ratio was taken as the correction coefficient.

This correction coefficient was then applied to the retail price to adjust it to the average purchase price. For 32 items such as “other noodles” and “other leafy vegetables”, which represent all foods in a certain category except for specific foods, neither the household survey nor the retail price statistics survey provided price information. For these food items, we used the average purchase price of food items in the same category. As an example, the purchase price of “other processed meat” was taken as the average purchase price of “ham”, “sausages”, and “bacon”. Also, for “shumai” and “cocoa/cocoa beverages”, while they are not items that bundle the same category into one, neither the household survey nor the retail price statistics survey provides price information. Therefore, we used the purchase price of similar food items as a substitute for the purchase price of these food items. The purchase price of “shumai” was substituted with the purchase price of “gyoza”, and the purchase price of “cocoa/cocoa beverages” was substituted with the purchase price of “coffee beverages”. In this manner, we calculated the average purchase price per 100 g and the purchase price for each food item. Then, by dividing the annual per-person expenditure on each food item by the price per 100 g, we calculated the annual purchase quantity (physical unit: 100 g) of each food item.

For each ingredient, we used the item that best corresponds to the 200 food items, and the average purchase price of that food item was used as the price of the relevant ingredient. The price of each dish is determined by the sum of the product the weight of each ingredient used in the recipe and the price of each ingredient. Unlike the calculation of the nutritional composition, the weight of each ingredient includes the weight of inedible parts. The price P per serving of each dish can be represented by the following Eq. 2.

2 $${\rm{P}}=\frac{{\sum }_{{\rm{i}}}{{\rm{Q}}}_{{\rm{i}}}\times {{\rm{P}}}_{{\rm{i}}}}{{\rm{S}}}$$

Here, P_i_ represents the average purchase price per weight of ingredient i [JPY/g].

### Estimation of the carbon footprint of recipes

To ascertain how much GHG emissions are necessitated by the consumption of each recipe overall, we estimated the carbon footprint at three stages: production and sales, cooking, and disposal. Similar to nutrients, in order to compare dishes, we divided the total carbon footprint by the number of servings per recipe to calculate the carbon footprint per serving. The carbon footprint E of each dish can be calculated using Eq. 3.

3 $${\rm{E}}=\frac{\left({{\rm{E}}}_{{\rm{p}}}+{{\rm{E}}}_{{\rm{c}}}+{{\rm{E}}}_{{\rm{d}}}\right)}{{\rm{S}}}$$

Here, E_p_ is the carbon footprint of the production and sales process [g], E_c_ is the carbon footprint of the cooking process[g], E_d_ is the carbon footprint of the disposal process, and S is the number of servings per recipe[g]. Firstly, to calculate the carbon footprint from supply-chain, which indicates the emission associated with the production and retail process, we applied the emission intensity generated from Embodied Energy and Emission Intensity Data for Japan Using Input-Output Tables (3EID)^55^, which originated from the EEIO method. To note, the consumer price is adopted. The difference and reason for adoption on consumer price is previously discussed in our previous study^56^. The carbon footprint E_p_ of the production and sales process of each dish can be calculated using Eq. 4.

4 $${{\rm{E}}}_{{\rm{p}}}=\sum _{{\rm{i}}}{{\rm{Q}}}_{{\rm{i}}}\times {{\rm{P}}}_{{\rm{i}}}\times {{\rm{I}}}_{{\rm{i}}}$$

Here, Q_i_ is the weight of ingredient i used in this dish [g], P_i_ is the average purchase price per weight of ingredient i [JPY/g], and I_i_ is the GHG emission unit of ingredient i [g/JPY]. However, for each dish, there may be food waste, such as leftovers and disposal. Since the ingredients that make up these food losses are not actually consumed, the carbon footprint emitted to produce and sell the ingredients of the food loss and the carbon footprint emitted to produce and sell the consumed ingredients must be considered separately. E_p_ is the carbon footprint of the production and sales process of all the ingredients necessary for a dish [g], including the carbon footprint of the food waste. To note, we would like to highlight that the methodology for estimating the carbon footprint from the production process has been extensively covered in prior studies. As for our approach to addressing the inconsistencies between intensities in the 3EID dataset and the consumption data, we referred to our previous dataset^57^. The calculation of the carbon footprint due to the food waste portion in the production and sales process will be described later in the section on the carbon footprint of the disposal process.

### Estimation of Energy Consumption Required for Each Cooking Method

Many preceding studies pertaining to the calculation of carbon footprint associated with cooking have established several menus, actually performed cooking, measured the consumption of gas or electricity used in the process, and then multiplied the carbon dioxide emission unit per energy type until it reaches the consumer, to calculate carbon dioxide emissions. However, such calculation methods necessitate actual cooking and measurement of each dish and can only calculate the carbon dioxide emissions of the dish in the set menu. On the other hand, Inaba *et al*.^58^ proposed a method for estimating CO_2_ emissions from home cooking, taking into account the thermal efficiency of cooking utensils and temperature rise according to each cooking method. In this study, we referred to the estimation method of Inaba *et al*. and calculated the carbon footprint of the cooking process based on the ingredients and cooking methods described in the recipe of the dish.

Cooking utensils are primarily divided into those that use electricity or gas (city gas or LP gas, this study assumes city gas for the calculation). Therefore, this study calculated by multiplying the CO_2_ emission unit per each power consumption or gas consumption used in cooking utensils. The electric cooking utensils targeted are microwaves, ovens, and rice cookers. When using electric cooking utensils, the recipe often clearly states the output and cooking time, such as “heat in a 500 W microwave for 5 minutes” or “bake in a 180 °C oven for 10 minutes”. From the output, the power consumption can be understood, and it can be calculated in conjunction with the cooking time to easily determine the power consumed during cooking.

On the other hand, when cooking with gas-fired cooking utensils such as gas stoves, the recipe may be clearly stated, such as “burn on high heat for 5 minutes”, or ambiguously stated, such as “boil until the vegetables are heated” or “fry until it turns fox-colored”. In the former case, by investigating the gas flow rate during high, medium, and low heat through experiments, if the cooking time is known, the approximate gas consumption can be estimated. However, in the latter case, it is very difficult to estimate in the same way because neither the size of the gas output nor the cooking time is shown.

Therefore, considering the thermal efficiency of gas cooking utensils, we estimated the amount of energy that needs to be transmitted to ingredients and water/oil according to each cooking method such as “boiling”, “grilling”, and “frying”. In this case, even if the size of the gas output and the cooking time are not known, if the ingredients used and their quantities and the cooking method are known, the required energy amount for cooking can be approximated.

The thermal efficiency of the gas cooking utensils was taken as the value obtained by dividing the amount of energy used to raise the temperature and evaporate food ingredients and water/oil by the amount of energy provided by gas required for actual cooking. This study, like Inaba *et al*.^58^ used the thermal efficiency calculated using the results of cooking experiments by Mizuno *et al*.^59^. Thermal efficiency varies depending on low, medium, and high heat, the presence or absence of a lid, and heating and boiling times. In this study, to make calculations simple, the thermal efficiency in the case of medium heat without a lid was used, regardless of the dish and cooking method.

### Microwave

When using an electric range, the amount of power consumption can be estimated from the output of the microwave and the cooking time. The actual power consumption is higher than the output of the microwave due to losses during the conversion from electricity to heat. The conversion efficiency was set by referring to the maximum output and power consumption of an actual product (Panasonic Corporation’s single-function range NEFL222). Therefore, the power consumption when cooking with a microwave can be estimated using Eq. 5.

5 $${\rm{Microwave}}\;{\rm{power}}\;{\rm{consumption}}\,({\rm{J}})=({{\rm{P}}}_{{\rm{m}}}\times \Delta {{\rm{T}}}_{{\rm{m}}}){\rm{/\eta m}}$$

Here, P_m_ is the high-frequency output of the microwave [W], ΔT_m_ is the cooking time of the microwave [sec], and ηm is the conversion efficiency of the microwave (0.71).

### Oven

The power consumption when using an oven can be estimated in the same way as a microwave. In the case of an oven, without considering the conversion efficiency, the power consumption of the oven function of an actual product (Panasonic Corporation’s oven range NE-UBS5A) was used for estimation. The power consumption can be estimated by multiplying the oven’s power consumption by the cooking time.

### Rice cooker

Not only for dishes where cooking with a rice cooker is stated in the cooking procedure of the recipe, such as rice and mixed rice, but also for dishes where rice is directly used as one of the ingredients, such as fried rice and rice bowl dishes, it was assumed that cooking was done using a rice cooker. When cooking with a rice cooker, the power consumption was set as constant, regardless of the amount of rice cooked in the dish. The necessary power consumption was based on an actual product (Panasonic Corporation’s IH Jar Rice Cooker SR-VSX101), and set for cooking time (156 Wh per cycle) and warming time (13.9 Wh per hour). When cooking with a rice cooker, it was set to keep warm for 2 hours, and the power consumption was the sum of the power consumption for one cycle of cooking and 2 hours of keeping warm, uniformly set at 183.8 Wh.

### Simmering

In the case of cooking methods that involve “simmering”, we assumed the method involves heating for a certain period of time after boiling water. In this case, the energy consumption required for cooking can be divided into three major parts: the energy required to boil water, the energy required to maintain the temperature of the water after boiling, and the energy required to heat the ingredients. These can be represented by Eqs. 6, 7, and 8.

6 $${\rm{Energy}}\;{\rm{consumption}}\,{\rm{until}}\;{\rm{water}}\;{\rm{boils}}\;({\rm{J}})=\frac{{{\rm{m}}}_{{\rm{w}}}\times {{\rm{c}}}_{{\rm{w}}}\times \Delta {{\rm{K}}}_{{\rm{b}}}}{{\rm{\eta h}}}$$

7 $${\rm{Energy}}\;{\rm{consumption}}\;{\rm{for}}\;{\rm{maintaining}}\;{\rm{the}}\;{\rm{temperature}}\;{\rm{of}}\;{\rm{the}}\;{\rm{water}}\;{\rm{after}}\;{\rm{boiling}}\,({\rm{J}})=\frac{{\rm{v}}\times {{\rm{S}}}_{{\rm{p}}}\times {{\rm{H}}}_{{\rm{w}}}\times \Delta {\rm{T}}}{{\rm{\eta b}}}$$

8 $${\rm{Energy}}\;{\rm{consumption}}\;{\rm{for}}\;{\rm{heating}}\;{\rm{ingredients}}\,({\rm{J}})=\frac{{\sum }_{{\rm{i}}}{{\rm{Q}}}_{{\rm{i}}}\times {{\rm{c}}}_{{\rm{i}}}\times \Delta {{\rm{K}}}_{{\rm{b}}}}{{\rm{\eta b}}}$$

Here, m_w_ is the amount of water used [g], c_w_ is the specific heat of water [J/(g·K)], ΔK_b_ is the temperature difference [K] from room temperature (20°C) to boiling point (100°C), ηh is the heat efficiency during heating, v is the evaporation amount of water per unit area of the pot [g/(min·cm^2^)], S_p_ is the bottom area of the pot [cm^2^], H_w_ is the latent heat of evaporation of water [J/g], ΔT is the heating time after boiling [min], ηb is the heat efficiency after boiling, Q_i_ is the amount of ingredient i used [g], and c_i_ is the specific heat of ingredient i [J/(g · K)]. When the cooking method is “simmering”, the amount of water used in the recipe is stated, so m_w_ uses the amount of water stated in each recipe. In addition, since ΔT is often not stated in the recipe, for simplicity of calculation, 2/3 of the total cooking time required for the recipe was used.

### Boiling

When the cooking method is “boiling”, similar to “simmering”, the energy consumption required for cooking can be divided into three parts: the energy required to boil the water, the energy required to maintain the temperature of the water after boiling, and the energy required to heat the ingredients. This can be determined by the sum of Eqs. 6, 7, and 8. When the cooking method is “boiling”, the amount of water used in the recipe is often not stated. When boiling ingredients, enough water is needed to cover the entire ingredients, so in this study, we referred to Kagome’s site^60^, and used a value of 5 times the weight of the ingredients for m_w_. Also, as with “simmering”, ΔT is often not stated in the recipe, and for simplicity of calculation, it was set uniformly at 10 minutes.

### Steaming

When the cooking method is “steaming”, similar to “simmering” and “boiling”, the energy consumption required for cooking can be divided into three parts: the energy required to boil the water, the energy required to maintain the temperature of the water after boiling, and the energy required to heat the ingredients. This can be determined by the sum of Eqs. 6, 7, and 8. Even when the cooking method is “steaming”, the amount of water used in the recipe is often not stated. The amount of water needed to steam the ingredients was set at 60% of the pot. In this study, the depth of the pot was set at 15 cm, and the amount of water needed to “steam” was set from the bottom area of the pot. Also, as with “simmering”, ΔT is often not stated in the recipe, and for simplicity of calculation, 2/3 of the total cooking time required for the recipe was used.

### Stir-fry

In the case of stir-frying on a gas stove, it is assumed that the heat balance between the gas stove and the bottom surface of cooking utensils such as pots and frying pans is the same, regardless of the method of cooking. However, when “grilling” with a frying pan, unlike when “boiling”, “blanching”, “steaming”, or “frying”, parts of the frying pan’s surface area that do not hold food or water/oil occur. The heat received by these parts from the gas stove is released into the atmosphere without being transferred to the food or water/oil. Taking into account such heat losses to the atmosphere, in order to calculate the real energy required to heat the food, the following correction factor was multiplied by the heat efficiency (See Eq. 9).

9 $${\rm{Correction}}\;{\rm{factor}}\;{\rm{for}}\;{\rm{frying}}\;{\rm{pan}}\,({\rm{\alpha }})=\frac{{{\rm{S}}}_{{\rm{s}}}}{{{\rm{S}}}_{{\rm{f}}}}$$

Here, S_s_ represents the area covered by food [cm^2^], S_f_ is the bottom area of the frying pan [cm^2^].

Using the correction factor for the frying pan, the energy consumption required when the method of cooking is “grilling” can be calculated using Eq. 10.

10 $${\rm{Energy}}\;{\rm{consumption}}\;{\rm{for}}\;{\rm{heating}}\;{\rm{the}}\;{\rm{food}}\,({\rm{J}})=\frac{{\sum }_{{\rm{i}}}{{\rm{Q}}}_{{\rm{i}}}\times {{\rm{c}}}_{{\rm{i}}}\times \Delta {{\rm{K}}}_{{\rm{f}}}}{{\rm{\eta h}}\times {\rm{\alpha }}}$$

Here, ΔK_f_ represents the temperature difference [K] of the food before and after stir-frying.

### Frying

For the cooking method of frying, the energy consumption required for cooking can be divided into two parts: the energy required to raise the oil to the necessary temperature, and the energy needed to heat the food ingredients. In the case of “frying”, in addition to these two types of energy, energy is also needed for the evaporation of water from the food. However, the amount of water evaporation from the food differs significantly depending on the food and the way it is fried, and it is difficult to calculate, hence this study did not take into account the energy consumption needed for the evaporation of water from the food due to frying. As a result, it is necessary to note that the calculated energy consumption needed for cooking may be an underestimate of the actual energy consumption. The amount of energy needed to raise the temperature of the oil can be calculated in the same way as the energy needed to bring water to boil in cases of “boiling”, “blanching”, and “steaming”. Moreover, the energy needed to heat the food is the same as the calculations for “boiling”, “blanching”, “steaming”, and “grilling”. The calculation formulas are shown in Eqs. 11 and 12.

11 $${\rm{Energy}}\;{\rm{consumption}}\;{\rm{required}}\;{\rm{for}}\;{\rm{raising}}\;{\rm{oil}}\;{\rm{temperature}}\,({\rm{J}})=\frac{{{\rm{m}}}_{{\rm{o}}}\times {{\rm{c}}}_{{\rm{o}}}\times \Delta {{\rm{K}}}_{{\rm{o}}}}{{\rm{\eta h}}}$$

12 $${\rm{Energy}}\;{\rm{consumption}}\;{\rm{for}}\;{\rm{heating}}\;{\rm{the}}\;{\rm{food}}\,({\rm{J}})=\frac{{\sum }_{{\rm{i}}}{{\rm{Q}}}_{{\rm{i}}}\times {{\rm{c}}}_{{\rm{i}}}\times \Delta {{\rm{K}}}_{{\rm{d}}}}{{\rm{\eta h}}}$$

Here, m_o_ represents the amount of oil used [g], c_o_ represents the specific heat of the oil [J/(g·K)], ΔK_o_ represents the temperature difference [K] from room temperature (20 °C) to frying temperature (180 °C), and ΔK_d_ represents the temperature difference [K] of the food before and after frying.

### Estimation of specific heat for each ingredient

In this section, we elucidate the specific heat of each food ingredient, which is crucial in calculating the carbon footprint of the cooking process of each dish. Obtaining data related to the specific heat of each ingredient used in the dishes targeted in this study was challenging. Therefore, barring the food ingredients whose specific heat is listed in the Thermal Property Handbook^61^, we utilized the specific heat calculated using estimation formulas.

Specific heat is minimally affected by heterogeneous structures, thus it can be calculated from the composition of water, protein, carbohydrates, and fats in food. The difference in specific heat between proteins and carbohydrates is small, hence they can be treated collectively as “solid matter.” Therefore, for low-fat foods that contain minimal fat, the specific heat can be calculated using the following Eq. 13, which M_W_ + M_S_ = 1.0:

13 $${\rm{c}}=\left(1.0{{\rm{M}}}_{{\rm{W}}}+0.2{{\rm{M}}}_{{\rm{S}}}\right)\times 4187$$

In this context, low-fat foods are those with a mass fraction of fat of 1% or less. Here, c represents the specific heat of the ingredient [J/(kg·K)], M_W_ represents the mass fraction of moisture, and M_S_ represents the mass fraction of protein and carbohydrates.

Fats have different phase transition temperatures depending on their composition and crystallinity. If high-fat foods contain fats with high phase transition temperatures, the apparent specific heat will include the latent heat of phase transition, resulting in higher specific heat than low-fat foods. Therefore, it is necessary to measure the specific heat of high-fat foods, but near room temperature, it can be estimated using the following Eq. 14, which M_W_ + M_F_ + M_S_ = 1.0:

14 $${\rm{c}}=\left(1.0{{\rm{M}}}_{{\rm{W}}}+0.5{{\rm{M}}}_{{\rm{F}}}+0.33{{\rm{M}}}_{{\rm{S}}}\right)\times 4187$$

Here, M_F_ represents the mass fraction of fat.

These estimation formulas apply when the sum of the mass fractions of water, protein, carbohydrates, and fats in food is 1.0. However, ingredients with a high proportion of ash, such as seasonings, may not be accurately estimated. Therefore, for ingredients with a mass fraction of ash of 0.1 or more, the specific heat is calculated using the following formula by Heldman and Singh^62^, where M_W_ + M_C_ + M_P_ + M_F_ + M_M_ = 1.0:

15 $$c=4187{{\rm{M}}}_{{\rm{W}}}+1424{{\rm{M}}}_{{\rm{C}}}+1549{{\rm{M}}}_{{\rm{P}}}+1675{{\rm{M}}}_{{\rm{F}}}+837{{\rm{M}}}_{{\rm{M}}}$$

Here, M_C_ represents the mass fraction of carbohydrates, M_P_ represents the mass fraction of protein, and M_M_ represents the mass fraction of ash.

### Carbon footprint of the cooking process

**Fig. 3:**
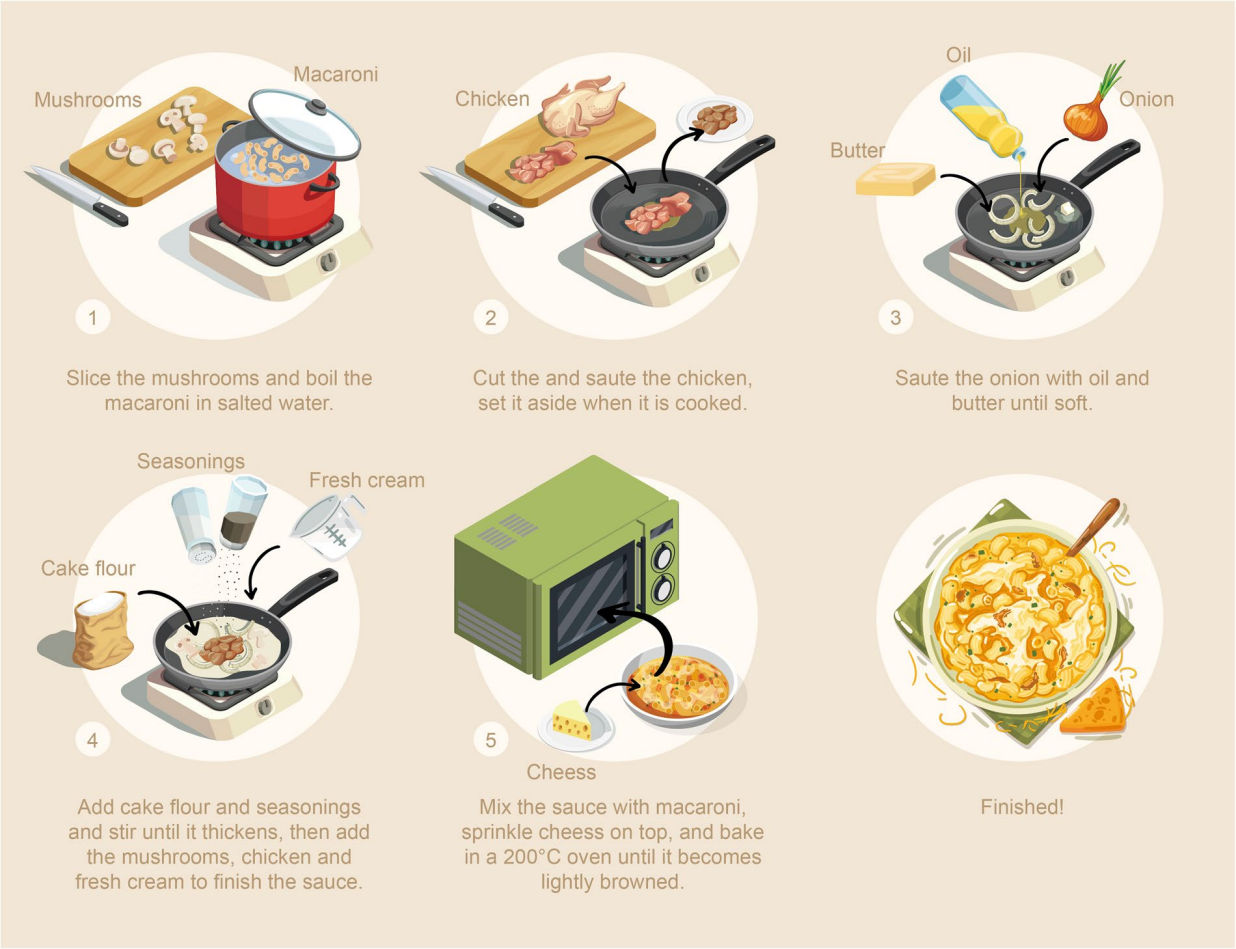
Flowchart of cooking macaroni gratin.

In this section, we discuss the calculation of the carbon footprint during the cooking process of various dishes, utilizing the previously estimated energy consumption values for each cooking method. However, most dishes are prepared through more than one cooking method. For instance, the preparation of macaroni gratin is outlined in the listed steps and depicted in Fig. 3, by illustrating the flowchart of the cooking process.

It becomes clear from the cooking procedure that macaroni gratin is prepared through various methods, including boiling, baking, simmering, and oven-cooking. For such dishes, it’s impossible to estimate the carbon footprint of the cooking process using a single cooking method. Furthermore, assuming that the heat energy absorbed by the ingredients during cooking remains constant when the final temperature is the same, it’s inappropriate to simply add up the carbon footprints calculated for each cooking method. Hence, in this study, for dishes cooked using multiple methods, we used the average of the carbon footprints calculated for each method.

However, in cases where both electric and gas cooking appliances are used for a dish, given that electric appliances are often used for preliminary preparations and final touches, the carbon footprint for the cooking process was taken as the average carbon footprint calculated for the gas cooking methods plus the carbon footprint from the electric appliances.

The carbon footprint from cooking with electric appliances was obtained by multiplying the estimated power consumption by the CO_2_ emission coefficient per kWh. The CO_2_ emission coefficient for electricity used here is based on the definitive value for the fiscal year 2020 (441g- CO_2_/kWh) provided by TEPCO Energy Partner, Inc.^63^. Similarly, the carbon footprint from cooking with gas appliances was obtained by multiplying the estimated energy consumption by the CO_2_ emission coefficient per MJ for city gas. The CO_2_ emission coefficient for city gas per MJ used here was calculated using the standard calorific value of city gas and the CO_2_ emission coefficient per cubic meter of city gas (49.9g- CO_2_/MJ) as announced by the Tokyo Gas Group^64^.

From the above, the carbon footprint E_c_ of each cooking process [g] can be calculated as follows:

16 $${{\rm{E}}}_{{\rm{c}}}={{\rm{W}}}_{{\rm{e}}}\times 441+\frac{{\sum }_{{\rm{i}}}^{{\rm{n}}}{{\rm{W}}}_{{\rm{g}},{\rm{i}}}}{{\rm{n}}}\times 49.9$$

Here, W_e_ is the total power consumption [kWh] used by electric cooking appliances, W_g,i_ is the energy consumption [MJ] of cooking method i using a gas cooking appliance, n is the number of types of cooking methods using a gas cooking appliance for each dish. Table 3 below provides a list of the values used for each constant in the calculation of the necessary energy consumption for each cooking method.

**Table 3:** Constants used in the calculation of the necessary energy consumption for each cooking method.

Constant Name	Details	Value
ηm	Conversion efficiency of microwave	0.71
ηh	Heat efficiency during heating	0.37
ηb	Heat efficiency after boiling	0.42
c_w_	Specific heat capacity of water [J/(g·K)]	4.20
H_w_	Latent heat of vaporization of water [J/g]	2250
c_o_	Specific heat capacity of oil [J/(g·K)]	1.96
ΔK_b_	Temperature difference from room (20 °C) to boiling temperature (100 °C) [K]	80
ΔK_f_	Temperature difference of food before and after baking [K]	100
ΔK_o_	Temperature difference from room (20 °C) to frying temperature (180 °C) [K]	160
ΔK_d_	Temperature difference of food before and after frying [K]	130
v	Evaporation of water per unit area of pot [g/(min·cm²)]	0.06
S_p_	Bottom area of the pot [cm²]	314
α	Correction factor of the frying pan	0.75

### Carbon footprint of the disposal process

In the consumption of food, a large amount of food waste is generated. Especially in Japan, most of the food waste generated at home is incinerated or landfilled, emitting a lot of CO_2_ in the process. Furthermore, the mass production of food waste has become a social issue in recent years, with food waste generated from homes alone amounting to approximately 2.61 million tons in 2019^65^. Energy is used and CO_2_ is emitted in all processes until food waste is generated, including production, storage, processing, transportation, consumption, and disposal. Thus, the carbon footprint associated with food waste has a significant impact on the carbon footprint of meals, making it a crucial aspect to consider. However, there is hardly any research on the carbon footprint of food waste generated from each dish.

Therefore, this study calculated the total amount of food waste generated from each dish by multiplying the food ingredients used in the dish by their respective waste rates and summing them. The CO_2_ emitted during the process of treating that food waste is regarded as the carbon footprint of the waste process.

Food waste is generated at various stages from production to actual consumption. First, in the production process, products that are unsuitable or have minor defects that do not affect consumption are discarded and not shipped as products. In the distribution and sales process, waste is generated due to attrition during transportation, in warehouses, and on store shelves, as well as unsold goods. Even after being sold to consumers, inedible parts such as skin, bones, and shells of seafood, eggshells, and vegetable skins and cores are removed and discarded during the pre-cooking process. In the edible part excluding the inedible part, the following three types of waste occur:

1. Leftovers: Food that was used as an ingredient in cooking or was eaten as is, but was left unfinished and discarded.
2. Direct Waste: Food that could have been used as an ingredient in cooking or could have been eaten as is, but was discarded as is due to the expiration of the best-before date, etc.
3. Excessive Removal: During cooking, parts that are edible are excessively removed while removing inedible parts are discarded. Specifically, this refers to parts that are removed in excess of the waste rate in the “ Japan-Standard Tables Of Food Composition” by the Ministry of Education, Culture, Sports, Science and Technology. It also includes edible parts that had to be removed due to spoilage.

In this study, we focus only on the food waste that occurs after the food reaches the consumer, including inedible parts, leftovers, direct waste, and excessive removal generated in each dish. Note that we collectively refer to food waste caused by leftovers, direct waste, and excessive removal, excluding inedible parts, as “food loss”. The weight of the inedible part of the food waste can be calculated by multiplying the weight of each food ingredient used in the recipe by the waste rate of each ingredient in the “Japan-Standard Tables of Food Composition”^52^. In the “2014 Food Loss Statistics Survey Report”, the amount of food used (edible part), and the amount of leftovers, direct waste, and excessive removal were surveyed for 26 food categories^66^. In this study, the waste rate of food loss was calculated using the results of the “2014 Food Loss Statistics Survey Report”, as showcased in Supplementary Table S1. For instance, this table indicates a food loss rate of 8.8% for ‘Vegetables’ and 2.2% for ‘Meat’ by physical unit. To reconcile the depth of the data with the need for clarity, we grouped 349 individual ingredients into 26 categories. The categorization rationale is based on similarities in food types, their consumption patterns, and related waste behaviors. Then, the waste rate of food loss for each food item was calculated using Eq. 17.

17 $${\rm{Food}}\;{\rm{loss}}\;{\rm{waste}}\;{\rm{rate}}\left({\rm{W}}\right)=\frac{{{\rm{W}}}_{{\rm{left}}}+{{\rm{W}}}_{{\rm{direct}}}+{{\rm{W}}}_{{\rm{excessive}}}}{{{\rm{U}}}_{{\rm{food}}}}\times 100 \% $$

Here, W_left_ is the amount of leftover waste [g], W_direct_ is the amount of direct waste[g], W_excessive_ represents the excessive removal waste amount [g], U_food_ is the food usage amount [g].

The total weight of food waste generated in each dish is the sum of the weights of food waste generated from inedible parts and food loss (leftovers, direct waste, excessive removal). In terms of food waste treatment, the CO_2_ emissions per unit of waste were calculated using the survey results of the “Study on Food Cycle” conducted by the Ministry of Agriculture, Forestry, and Fisheries. Food waste has various treatment methods such as energy use through fertilizer or methanation, solid waste fuel, and incineration (including heat recovery). In Japan, considering that most of the food waste generated at home is incinerated and landfilled, this study calculated the CO_2_ emissions when treating food waste (raw garbage) by incineration (without power generation). Burning food waste produces carbon dioxide, but it is also possible to consider it as carbon neutral because the overall balance of emission and absorption is maintained by absorbing carbon dioxide from the atmosphere during the growth process of plants, without affecting the increase or decrease of carbon dioxide. However, this study focuses on the CO_2_ emissions generated during the treatment process. In the case of incineration, emissions during each stage from when food waste is generated, collected, transported, incinerated, and the incineration residue is landfilled, are targeted. The CO_2_ emissions per 1000 kg of raw garbage at each stage when incinerating food waste are shown in Table 4.

**Table 4:** CO_2_ Emissions per 1000 kg of Raw Garbage at Each Stage in the Incineration of Food Waste.

Stages	CO_2_ Emissions (kg)
Collection	24
Incineration	73
Waste Transportation	3.7
Landfill	5.7
Total	106.4

The carbon footprint E_d_ of the waste process [g] generated by each dish can be calculated as follows using Eq. 18:

18 $${{\rm{E}}}_{{\rm{d}}}={\sum }_{{\rm{i}}}\left({{\rm{Q}}}_{{\rm{i}}}\times {{\rm{D}}}_{{\rm{i}}}+{{\rm{Q}}}_{{\rm{i}}}\times \left(1-{{\rm{D}}}_{{\rm{i}}}\right)\times {{\rm{W}}}_{{\rm{i}}}\right)\times 0.1064$$

Where Q_i_ is the weight of ingredient i [g], D_i_ is the waste rate of the inedible part of ingredient i, and W_i_ is the waste rate of food loss in the food item classification corresponding to the ingredient i.

Food waste due to food loss is different from the waste of inedible parts, and can be reduced by efforts and ingenuity. Therefore, we can consider that the amount of food ingredients is unnecessarily produced only by the amount discarded by food loss, and the carbon footprint of the production and sales process of the food ingredients of the food loss part would not occur if there were no food loss. In this study, we estimated the carbon footprint of the production and sales process of the food loss part of each dish in order to distinguish it from the carbon footprint of the production and sales process of the actual consumed ingredients.

The carbon footprint of the production and sales process of the food loss part among the carbon footprints of the production and sales process of each recipe, E_p,foodloss_, was estimated by Eq. 19.

19 $${{\rm{E}}}_{{\rm{p}},{\rm{foodloss}}}={\sum }_{{\rm{i}}}{{\rm{Q}}}_{{\rm{i}}}\times \left(1-{{\rm{D}}}_{{\rm{i}}}\right)\times {{\rm{W}}}_{{\rm{i}}}\times {{\rm{P}}}_{{\rm{i}}}\times {{\rm{I}}}_{{\rm{i}}}$$

Here, P_i_ is the average purchase price per weight of ingredient i [JPY/g], and I_i_ is the emission unit of ingredient i [g/JPY]. The carbon footprint of the production and sales process of the actually consumed ingredients is obtained by subtracting the carbon footprint of the production and sales process of the food loss part from the carbon footprint of the production and sales process of all the ingredients, and can be calculated as E_p_ − E_p,foodloss_.

## Data Records

The comprehensive recipe nutrition and environmental impact database is stored as a detailed spreadsheet, accessible on FigShare^67^. The dataset is methodically arranged, encapsulating an integrated evaluation based on EEIO analysis combined with a hybrid LCA approach. This was designed to quantify both the carbon footprint throughout the food supply chain to cooking and waste and the distinct nutritional values for specific recipes. The dataset comprises 47 fields, which can be categorically distilled into four distinct domains: General Information, Nutritions and Keywords, Detailed Ingredient and Nutritional Information, and Environmental Impact Analysis. To enhance the user experience and facilitate pinpoint precision during data retrieval, Table 5 elucidates specific field names accompanied by their associated data descriptors. Furthermore, the ‘nutrients’ section offers a succinct breakdown, encapsulating metrics such as Energy, Fat, Carbohydrates, Zinc, Folic acid, Protein, Total fiber, Vitamins (A, C, E), Calcium, Iron, Potassium, Magnesium, Saturated fat, Cholesterol, and Salt equivalent, all of which are provided within the database.

**Table 5:** Database Structure and Description.

Category	Field Name	Meaning	Category	Field Name	Meaning
General Information	recipe_id	Unique identifier for each recipe.	Nutrition and Keywords	cliped_count	The number of times the recipe has been clipped or saved.
	recipe_name	The name of the recipe.		search_word	Relevant search terms linked to the recipe.
	img_url	URL of the recipe image.	Detailed Ingredient and Nutritional Information	ingredients	Detailed list of ingredients used.
	author_type	The type of author who provides the recipe.		modified_ingredients	Variations or modifications to the standard ingredients.
	author_name	Name of the author of the recipe.		nutrients	Detailed nutritional breakdown.
	published_datetime	Date and time the recipe was published.	Environmental Impact Analysis	GHG_production	GHG emissions during production.
	modified_datetime	Date and time the recipe was modified.		price	Cost associated with the recipe or its components.
	recipe_description	Brief description of the recipe.		disposal_amount	Amount of waste produced.
	servings	Number of servings the recipe yields.		GHG_disposal	GHG emissions during waste disposal.
	cooking_time	Duration required to cook the recipe.		GHG_cooking	GHG emissions during the cooking process.
	ingredients_inline	Ingredients listed in a single line.		GHG_total	Total GHG emissions associated with the recipe.
	instructions	Step-by-step cooking instructions.		dish	The type or category of the dish.
	recipe_cuisine	Type of cuisine the recipe belongs to.		ingredients_category	Category classification of ingredients.
Nutrition and Keywords	nutrition	General nutritional description of the recipe.		category	General category of the recipe.
	keywords	Keywords associated with the recipe.		cooking_method	Method used for cooking.

## Technical Validation

### Data validation

In order to validate our dataset, meticulous attention is paid to the nutritional content of various recipes, by drawing comparisons with authoritative sources. We incorporated data from the United States Department of Agriculture (USDA)^68^, FAO/INFOODS Food Composition Databases^69^, and FAO ASEAN Food Composition Database^70^ into the validation process. Since we have calculated the nutritional content of each dish in this database, the validation process contains a meticulous alignment of our dataset with the corresponding ingredients present in the FAO and USDA databases. This alignment encompassed a mapping of ingredients, quantities, and seasonings, enabling a comparison of nutritional values across the three datasets. Specifically, we extracted all 97 kinds of components in all 388 kinds of dishes, then we calculated the total content of 12 kinds of nutrition in all 97 kinds of components.

**Fig. 4:**
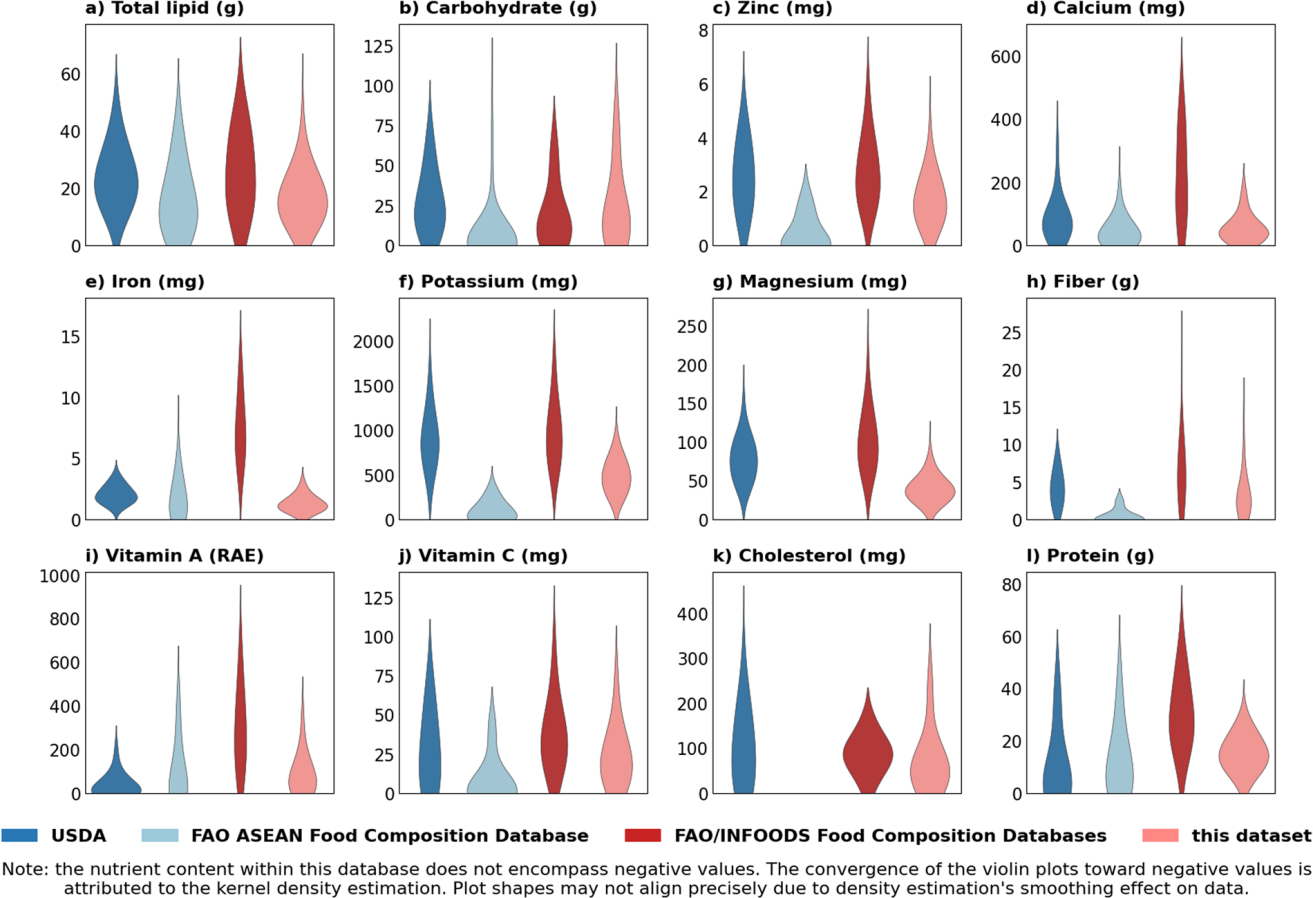
Comparative Nutritional Distribution Analysis of 388 Recipes from Different Data Sources.

Figure 4 presents the results of a rigorous validation procedure, illustrating the distribution of 12 nutritional constituents across a sample of 388 recipes through the medium of violin plots. The illustration takes the form of violin plots, where the horizontal axis designates the data sources: USDA (in deep blue), FAO ASEAN Food Composition Database (in pale blue), FAO/INFOODS Food Composition Databases (in pale pink), and the current dataset (in pale red). Since the FAO ASEAN Food Composition Database does not provide detailed information on the nutritional content of Magnesium and Cholesterol, the corresponding violin figures are left blank. Notably, the nutritional evaluations from our dataset align well with the other databases, as evidenced by the congruence across all 12 nutritional categories. This empirical evidence affirms the credibility of the nutritional data housed within our database.

In the quantification process of each recipe’s carbon footprint, we utilized average unit price for each food item, expressed per g/JPY. This representation, while providing a baseline perspective, failed to capture the inherent price variability influenced by factors such as differing consumer demographics, particularly across various income groups. Recognizing this limitation, we conducted an analysis to include price fluctuations. For 347 food items listed in the Family Income and Expenditure Survey, we discerned the minimum and maximum unit prices contingent on purchases from diverse income households within Japan. This variability in prices led to a recalibration of the food ingredient expenses for each recipe, which consequently refined our carbon footprint estimations related to the production phase. Our calculations unveiled an average uncertainty range of 41.47 g CO2 across the recipes, with an average relative uncertainty of 9.24% for GHG emissions during the production process, and 8.41% for total GHG emissions. Detailed information regarding the uncertainty analysis is provided in an excel file sheet in our dataset, titled ‘Uncertainty analysis’. This enhanced analysis underscores the tangible impact of ingredient price fluctuations on carbon footprint assessments. Notwithstanding these advancements in our uncertainty estimations driven by price variances, we acknowledge that challenges persist in estimating uncertainties related to other aspects in the absence of granular personal survey data.

## Usage Note

The calculation of this dataset involves data from multiple data sources, including the 3EID dataset and some public data from the Japanese Government Statistics. Researchers and experts can extract information from any database by themselves, and follow our processing steps to achieve data cleaning and enhancement. Non-data processing experts can directly use the summary excel file “recipe_data.xlsx”. Possible research applications of this dataset include identifying a) differences between cuisine styles, b) differences in diet patterns, c) diet costs and environmental impact, etc. In terms of policy and practice, integrating this dataset with health and environmental policies can provide support to governments and organizations to develop healthier and sustainable dietary guidelines and policies. Despite the meticulous research process, we acknowledge the absence of a detailed uncertainty analysis, particularly regarding the carbon footprint embodied in the production process. Therefore, when factoring in the entire food supply chain of ingredients, carbon footprints from the production stage might potentially overshadow that from cooking and disposal processes. We admit that our difficulty in acquiring personal survey data presents challenges in a detailed statistical analysis and urges users to exercise caution when interpreting the results considering potential uncertainties associated with the data. Users interested in using the methodological framework or data presented here are requested to cite this manuscript.

### Supplementary information

Supplementary Table S1. Household food loss rate in fiscal year 2014

## Data Availability

The code for data validation is open-source and available at the GitHub repository https://github.com/LiqiaoHuang/Recipe_dataset.git.
