# Supplementary material for: Carbon footprint and embodied nutrition evaluation of 388 recipes
Source: Sci Data. 2023 Nov 10;10:794. doi: 10.1038/s41597-023-02702-1 (PMC10638372; doi:10.1038/s41597-023-02702-1)
Supplement: Supplementary file 1 — Supplementary Table S1. Household food loss rate in fiscal year 2014 [file 41597_2023_2702_MOESM1_ESM.docx]

**Table S1**. Household food loss rate in fiscal year 2014

| Name | Average food loss rate (%) |
| --- | --- |
| Total | 3.7 |
| Grain | 1.3 |
| Starch | 3.3 |
| Beans | 1.4 |
| Vegetables | 8.8 |
| Potato | 10.9 |
| Green And Yellow Vegetables | 8.1 |
| Mushrooms | 8.6 |
| Fruits | 8.6 |
| Meat | 2.2 |
| Eggs | 2.0 |
| Milk And Dairy Products | 0.8 |
| Fish And Shellfish | 5.8 |
| Fresh Seaweed | 1.7 |
| Sugar | 2.2 |
| Oils And Fats | 0.2 |
| Condiments | 3.7 |
| Cooked Food | 1.8 |
| Rice | 1.6 |
| Bread | 1.2 |
| Can / Bottle | 1.5 |
| Frozen Products | 0.8 |
| Retort Food | 1.9 |
| Side Dishes, Processed Products, etc. | 1.9 |
| Confectionery | 1.3 |
| Beverages | 0.6 |
